# A bioinformatics workflow for detecting signatures of selection in genomic data

**DOI:** 10.3389/fgene.2014.00293

**Published:** 2014-08-26

**Authors:** Murray Cadzow, James Boocock, Hoang T. Nguyen, Phillip Wilcox, Tony R. Merriman, Michael A. Black

**Affiliations:** ^1^Department of Biochemistry, University of OtagoDunedin, New Zealand; ^2^Virtual Institute of Statistical GeneticsRotorua, New Zealand; ^3^Department of Mathematics and Statistics, University of OtagoDunedin, New Zealand; ^4^New Zealand Forest Research Institute LtdRotorua, New Zealand

**Keywords:** signatures of selection, genomics, genome-wide, analysis pipeline

## Abstract

The detection of “signatures of selection” is now possible on a genome-wide scale in many plant and animal species, and can be performed in a population-specific manner due to the wealth of per-population genome-wide genotype data that is available. With genomic regions that exhibit evidence of having been under selection shown to also be enriched for genes associated with biologically important traits, detection of evidence of selective pressure is emerging as an additional approach for identifying novel gene-trait associations. While high-density genotype data is now relatively easy to obtain, for many researchers it is not immediately obvious how to go about identifying signatures of selection in these data sets. Here we describe a basic workflow, constructed from open source tools, for detecting and examining evidence of selection in genomic data. Code to install and implement the pipeline components, and instructions to run a basic analysis using the workflow described here, can be downloaded from our public GitHub repository: http://www.github.com/smilefreak/selectionTools/

## Introduction

With the increased availability of whole-genome genotype data, it is possible to identify regions of the genome that exhibit evidence of having been subjected to selective pressure (e.g., Sabeti et al., 2002, 2007). While these “signatures of selection” can help to shed light on the evolutionary pressures experienced throughout history, they have also been shown to be associated with regions of the genome that are enriched for genes involved in cultural differentiation and complex disease in humans (Laland et al., 2010; Lappalainen et al., 2010) and traits of adaptive and/or commercial significance in plant and animal species. Examples include forest trees (see reviews by Gonzalez-Martinez et al., 2011; Neale and Kremer, 2011), wheat (Cavanagh et al., 2013), horses (Gu et al., 2009), sheep (Moradi et al., 2012), and domesticated dairy cattle (e.g., Qanbari et al., 2011). As a result, methods for detecting evidence of selection also provide a mechanism for highlighting genomic regions that may be associated with biologically important traits.

Recently Pybus et al. (2014) described the “Selection Browser 1.0,” a web-based tool for investigating selection in the human genome, based on a subset of data available from the 1000 Genomes Project (The 1000 Genomes Project Consortium, 2010). This resource delivers easy and intuitive access to pre-computed results from a number of tests for selection, applied to the available data, and thus provides an excellent example of the type of selection-specific information that can be extracted from low-coverage resequencing studies. For researchers wishing to investigate selection in other human cohorts or populations (or other organisms), however, a non-trivial amount of data manipulation and subsequent computation is required in order to extract this type of information from the available data.

Although detection of putative selective pressure offers a useful approach for identifying regions of interest in the genome, a number of steps are required to move from genome-wide (re)sequence or genotype data (e.g., as can easily be obtained using high-throughput microarray-based or sequencing technologies) to identifying specific genomic regions that exhibit evidence of having been under selection. The steps required reflect to some extent the series of advances that have been made in genomics technologies in recent years, with traditional file formats and software requiring manipulation and translation as part of the analysis workflow. While the process is not complex, for researchers unfamiliar with the required tools and data formats, the path from genotypes to signatures of selection can be a difficult one.

Here we provide a brief overview of a relatively simple workflow for taking high-density genotype data, and using it to identify evidence of selective pressure in regions of the genome. This pipeline is applicable to any diploid species where genome-wide (re)sequence and/or genotypic data are available (e.g., genomic/transcriptomic sequencing, whole genome SNP arrays), along with an ancestral reference genome and either a genetic or physical map.

## Methods for detecting evidence of selection

The tools used to detect evidence of selection are dependent on the nature of the selective signature being investigated, which itself depends on the time scale over which the selection occurred (Sabeti et al., 2006). Traditionally the F_ST_ statistic has been a popular choice for investigating selection, utilizing differences in allele frequency between populations to infer selective pressure in one population relative to the other, and allowing detection of potential selection occurring in the range 50,000 to 75,000 years prior for human populations (Sabeti et al., 2006), equivalent to approximately 2000 to 3000 generations. A thorough review of the use of the F_ST_ statistic is provided by Holsinger and Weir (2009), with recent modifications able to account for genotypic uncertainty associated with more modern technologies (Fumagalli et al., 2013). Differences in F_ST_ estimates have been discussed by Bhatia et al. (2013), who examined the effect of choosing different estimation methods and SNP sets on estimates of F_ST_. Both of these aspects were found to impact F_ST_ estimates, and the authors recommend that care be taken in the choice of both the estimators, and the SNPs being used.

Analysis of the reduction in genetic diversity provides another approach to examining selection, allowing the detection of possible “selective sweeps” which have resulted in regions where an allele conferring a selective advantage has risen in frequency in a population, carrying other variants in linkage disequilibrium to similarly increased frequencies, and thus reduced levels of diversity at that genomic locus. Tajima's D statistic (Tajima, 1989) provides a popular method for identifying such regions (see reviews by Sabeti et al., 2006; Barrett and Hoekstra, 2011; Iskow et al., 2012). More recently, modified methods have been developed to account for ascertainment bias in SNP microarrays (Ramírez-Soriano and Nielsen, 2009). Tajima's D is suitable for detecting evidence of positive selection in human populations occurring within the past 250,000 years (Sabeti et al., 2006) or approximately 10,000 generations, and operates by identifying an excess of low-to-intermediate frequency variants. Another commonly used measure is Fay and Wu's H (Fay and Wu, 2000) which is useful for detecting evidence of more recent positive selection (<80,000 years: Sabeti et al., 2006, or approximately 3000 generations), particularly for intermediate-high frequency variants, and thus complements Tajima's D and other methods (see Fay and Wu, 2000).

The advent of genome-wide genotyping technologies has facilitated the creation of whole genome haplotype maps, exemplified by the efforts of the HapMap Consortium (International HapMap Consortium, 2003) for studying natural variation in humans, and with more recent initiatives extending this approach to other species including bovine (The Bovine HapMap Consortium, 2009), maize (Gore et al., 2009), and rice (Huang et al., 2010). Analysis of haplotypes provides another mechanism for identifying evidence of selection, with a number of methods utilizing the Extended Haplotype Homozygosity (EHH) concept (Sabeti et al., 2002). One of the more popular of these approaches is the Integrated Haplotype Homozygosity Score (iHS) methodology, which provides a standardized measure of the decay in EHH around a point (e.g., a SNP) from the derived allele relative to the ancestral allele (Voight et al., 2006). Regions of slowly decaying haplotype homozygosity in the derived allele (i.e., longer than expected haplotypes, relative to the ancestral allele) are thus indicative of selection at that locus.

Underlying all of these tools are a number of demographic assumptions about the population(s) of interest, which must be considered when attempting to detect evidence of selection. In particular, for each method it is assumed that the existence of selective pressure is the most likely explanation for the generation of a statistically significant result. If present, other potential modifiers of variant frequency in a population can cause these tests to generate significant results, even in the absence of selection. Specific examples include: random drift, population bottlenecks, and population expansion, all of which can modify variant and haplotype frequencies in ways similar to selection. Some knowledge of the evolutionary history of the populations under study is therefore essential when considering the results generated when testing for evidence of selection.

## Applying selection tools to genomic data

A number of software tools exist which implement the various methods described above for detecting evidence of selection. In order to use a specific tool, however, the data in question must be in an appropriate format. Both the F_ST_ statistic, and Tajima's D, can be calculated using standard genotype data (e.g., SNP genotypes per individual). The iHS methodology, however, requires the use of haplotypes, and thus genotype data obtained from heterozgoygous populations must be phased prior to calculation of iHS. Various software applications exist for phasing genotype data (e.g., see Browning and Browning, 2011), although large differences in accuracy and speed exist between the various algorithms (Williams et al., 2012). Traditionally the Beagle algorithm has been a popular choice for phasing (Browning and Browning, 2007), although a number of recently developed algorithms are offering increased speed and accuracy (Williams et al., 2012; Delaneau et al., 2013).

Once phasing is complete, the rehh package (Gautier and Vitalis, 2012) provides a relatively simple interface for implementing various EHH-based analyses (including iHS) within the R computing environment (R Core Team, 2014). Additionally, rehh provides tools for visualizing loci under selection, such as haplotype bifurcation plots (Sabeti et al., 2002).

## Bioinformatics workflow

In order to simplify the process of analysing genomewide genotype data to identify selection signatures, we have developed a collection of scripts that implement the various tools described above. These scripts are publicly available via GitHub, and include instructions for installation and usage, as well as a detailed manual containing a worked example using a downloadable data set. The following sections describe the analytical processes implemented in the workflow.

### Data processing and analysis via command line tools

The analysis pipeline described here runs within a standard Linux operating system (in our case, Ubuntu 13.04, although almost any Linux-based system would be suitable), and requires the installation of a relatively small number of software tools (Table 1). The starting point of the analysis is a variant call format (VCF) file of the genotype data of interest (Danecek et al., 2011). This is a text file containing (at a minimum) information about variant positions, reference and alternative bases, and genotypes per sample. In order to permit calculation of measures comparing selection between multiple populations (e.g., F_ST_), samples from at least two populations are required to be present in the VCF file. Additionally, a file listing the subject identifiers for each population is also required, along with a genetic map of the chromosome(s) of interest in either SHAPEIT (Delaneau et al., 2013) or PLINK (Purcell et al., 2007) format. As a genetic map may not contain distances for all markers present in the VCF file, the genetic distance is inferred by linear interpolation (Nievergelt et al., 2004). If a genetic map is not available for the organism under study, a physical map (e.g., a reference genome) can be substituted, an approach that has recently been used in cattle (Gautier and Naves, 2011). Alternatively, if a representative sample of the species of interest is available, the LDHat software (McVean, 2014) can be used to generate recombination rate estimates, allowing conversion of physical distance to genetic distance, as was done in a recent analysis in *Arabidopsis* (Meijón et al., 2013).

**Table 1:** **Software tools used in the selection analysis workflow**.

**Application and version**	**Use in workflow**	**Website**
R ≥ v3.0	rehh	http://www.r-project.org
Perl ≥ v5.0	Vcftools modules vcf-subset and vcf-merge	http://www.perl.org/
Python ≥ v2.6	Running pipeline, haps file filtering and ancestral allele annotation	https://www.python.org/
rehh v1.11	Calculating iHS (and other EHH-based measures)	http://cran.r-project.org/package=rehh
vcftools v1.11	Conversion of VCF genotype data to PLINK format, and calculation of F_ST_ and Tajima's D	http://vcftools.sourceforge.net
SHAPEIT v2.r790	Phasing the PLINK formatted data to produce phased haplotype file	http://www.shapeit.fr
Beagle v4 r1274	Phasing un-phased VCF data to produce phased haplotype file	http://faculty.washington.edu/browning/beagle/beagle.html
PLINK v1.07	Remove SNPs with too many genotypes missing, filter on HWE and MAF	http://pngu.mgh.harvard.edu/~purcell/plink
tabix/bgzip v0.2.5	Required to get VCF into compressed and indexed format for vcftools	http://samtools.sourceforge.net/tabix.shtml
Multicore v0.1-7	R multicore package used to parallelise rehh runs	http://cran.r-project.org/web/packages/multicore/index.html
impute2 v2.3.1	Imputing genotypes from phased haplotype data	http://mathgen.stats.ox.ac.uk/impute/impute_v2.html
Pyfasta v0.5.2	Required to process ancestral fasta files	https://github.com/brentp/pyfasta
PyVcf v0.6.0	Required to process VCF files in python scripts	https://github.com/jamescasbon/PyVCF
Variscan v2.0.3	Calculation of Fay and Wu's H	http://www.ub.edu/softevol/variscan/

### Analysis of a single population

For a population VCF file that contains phase information, indels are first removed using the *vcftools* software (Danecek et al., 2011), as ancestral allele data are only available for SNP genotypes. The VCF is then converted to the Haps format (phased haplotypes: SNP genotypes per haplotype, per individual).

For a population VCF file without phased information, the file is converted to *PLINK* format (ped/map files) using *vcftools*. The Ped file contains relatedness information (if any) between subjects, affection status (e.g., for human case/control studies), and genotype data, while the “Map” file contains the genomic location of each variant (e.g., SNPs). PLINK is then used to filter the data based on multiple criteria (missingness, minor allele frequency, Hardy Weinberg Equilibrium, indels), and phasing is performed via SHAPEIT v2 (Delaneau et al., 2013) to produce a “Haps” file of phased haplotypes (SNP genotypes per haplotype, per individual) and a “Sample” file (genotype-specific information). Alternatively (or if a physical map is used), *Beagle* can be used to phase the data (Browning and Browning, 2007). If imputation is required, then *impute2* (Howie et al., 2009) is used, followed by a second round of indel filtering (to remove any indels introduced by the imputation process).

The phased data are annotated with ancestral allele information (via a custom Python script). These data are then analyzed in R (R Core Team, 2014) where the R package rehh (Gautier and Vitalis, 2012) is used to calculate EHH, and integrated EHH (iES).

### Analysis of multiple populations

If genotype data from multiple populations are available, then the data from the VCF file are used to calculate F_ST_ between each pair of populations using *vcftools*. F_ST_ is calculated using both the method of Weir and Cockerham (1984), and the method developed as part of the HapMap project (International HapMap Consortium, 2005). The genotype data are then split into per-population VCF files, and the analysis of each population proceeds as described above (“Analysis of a single population”), producing filtered phased data, and EHH and iHS values. Calculation of iHS requires knowledge of the ancestral allele relating to the SNP of interest. For human data, this information was traditionally generated through direct comparison of DNA to that of a close phylogenetic relative, such as the chimpanzee. More recently, however, phylogenetic trees have been used to derive ancestral alleles in humans, based on DNA sequence data from related species. The ancestral information used here comes from the ancestral FASTA files provided by the 1000 Genomes Project and derived 6-way Enredo-Pecan-Ortheus (EPO) alignment (Paten et al., 2008a,b) from the Ensembl Compara 59 database (Flicek et al., 2012). For non-human species, a FASTA file containing ancestral allele information is required. These are also available via Ensembl for some animal species (along with the 6-way EPO alignment for humans) from: http://www.ensembl.org/info/genome/compara/analyses.html

Alternatively, the EPO pipeline can be used locally to generate an ancestral reference, or a two-way alignment can be performed between the genome of interest, and that of a species with which a recent common ancestor is shared. This approach has previously been applied to human and chimpanzee by Voight et al. (2006) and to *Arabidopsis thaliana* and *A. lyrata* by Meijón et al. (2013). The script used here can annotate either a phased haps file or a phased VCF file using the ancestral allele information. Finally, for each pair of populations, Rsb (the standardized ratio of iES from two populations) is calculated using the rehh package in R (Voight et al., 2006; Tang et al., 2007).

### Visualizing the outputs—investigating selection at the human lactase gene locus as an example

Once the various measures of selection have been calculated in a genotype data set from one or more populations, it is helpful to visualize the results. As mentioned above, the public GitHub repository for the pipeline includes a worked example of running the code on a human data set. The data set used relates to a subset of genotype data from chromosome 2 of the human genome, derived from data downloaded from the 1000 Genomes Project. Of interest is the region around the gene encoding *lactase* (LCT - HG19 chr2: 136,545,410–136,594,750), which has shown evidence of selection over the past 5000–10,000 years (Bersaglieri et al., 2004). The CEU (European) and YRI (Yoruban) populations were used for the analysis here, comprising 85 and 88 samples respectively.

**Figure 1:**
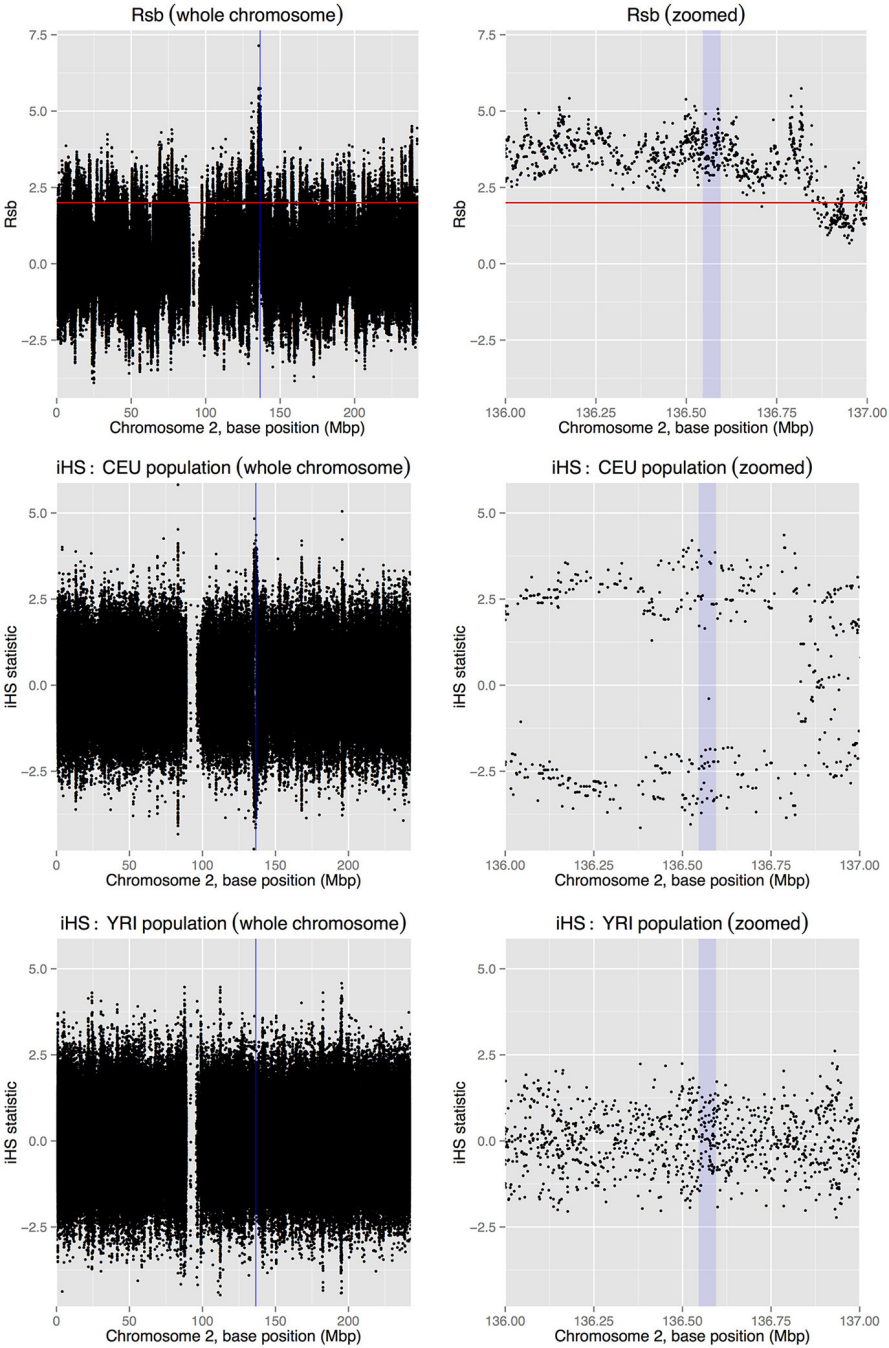
**Plots of Rsb (top row) and iHS (middle and bottom rows) values across chromosome 2 (whole chromosome in the left column, and the region around the LCT gene in the right column) based on 1000 Genomes Project data for the CEU and YRI populations**. Blue vertical lines/boxes on the plots indicate the location of the LCT gene, and the red horizontal lines denote a *p*-value of less than 5% for any Rsb value above the line. The marked deviation of iHS away from zero in the CEU population provides evidence for the region around the LCT gene having been under selective pressure in the past. In contrast, there is no such evidence in the YRI population, which is also communicated by the Rsb statistic, which examines the relative evidence for selection in the two populations, here indicating that there is stronger evidence for this region having been under selective pressure in the CEU cohort.

The analysis pipeline produced results for the following statistics: F_ST_, Rsb, iHS, Fay and Wu's H, and Tajima's D. A window size of 30 Kbp was used for calculating F_ST_ and Fay and Wu's H (with a sliding window of 3 Kbp for the latter), and a 3 Kbp window was used for Tajima's D. Figure 1 contains plots of Rsb and iHS for the CEU and YRI populations (chromosome-wide, and zoomed-in around the LCT gene), generated in R using the ggplot2 package (Wickham, 2009). The plots show clear evidence for differing degrees of selective pressure in the LCT gene between the CEU and YRI populations (i.e., selection in the CEU population), supporting previous observations in the literature (e.g., Bersaglieri et al., 2004). Not all of the measures of selection generated by the pipeline support this conclusion, however, with similar plots for F_ST_ (Figure S1), Tajima's D (Figure S2), and Fay and Wu's H (Figure S3), providing little evidence of selection in this region. These results (which agree with those for LCT available via the “Selection Browser 1.0” application of Pybus et al., 2014) highlight the importance of utilizing multiple measures for investigating selection, with different methodologies producing quite different results when applied to the same data. This again reinforces the fact that the various methods are utilizing different patterns of genetic variation to identify evidence of selection.

## Discussion

Here we present a simple workflow, and an associated collection of shell and R scripts, for identifying signatures of selection in diploid organisms. The workflow allows researchers to start from a collection of genome-wide genotype data for multiple individuals, and use a collection of freely available software tools to identify regions that exhibit evidence of having undergone selection. A range of tools have been developed for specific analyses of smaller data sets (e.g., Librado and Rozas, 2009; Delport et al., 2010), however the workflow presented here has the ability to analyze large data sets using multiple analytical methods to detect evidence of selection. An additional benefit of this tool set is our incorporation of parallelization capability into some of the tools to speed up analyses. These include rehh, vcf-subset, SHAPEIT, and IMPUTE2. We have also included a version of rehh which invokes the R multicore package (Urbanek, 2011) to allow utilization of multiple CPU cores. Other tools could potentially be parallelized should they become bottlenecks in analytical performance in larger data sets. The example analysis of chromosome two presented here required approximately 12.5 h of computation, running on 10 cores of a recent multicore linux server.

The methods described here fall broadly into three categories: frequency-based methods (Tajima's D and Fay and Wu's H), linkage disequilibrium-based methods (Rsb and iHS), and population differentiation-based methods (F_ST_), as reviewed by Vitti et al. (2013). By using each of these approaches, the differing characteristics of each method provide users with the ability to identify patterns of selective pressure arising in distinct contexts. As noted earlier, the time scale over which selection has occurred has a major impact on the ability of each method to detect evidence of its presence, with the frequency-based and population differentiation-based methods best suited to detecting events occurring further in the past. This is because these methods rely on the accumulation of additional mutations around the causal variant. In situations where the fitness advantage of the selected variant is small (particularly if it is recessive), then the time taken for the selected variant to rise to a detectable frequency in the population will be much longer, thus reducing the power of these methods. In situations where a new mutation (or a previously neutral variant encountering an environment of altered selective pressure) provides a fitness advantage and rises in frequency in the population without achieving fixation, linkage disequilibrium-based approaches provide increased power for detecting evidence of selection (Ferrer-Admetlla et al., 2014).

The differences in the results produced here for the human LCT example reflect the underlying methods of detection employed by each of the approaches, with time scale likely having a major impact. The three methods which found no evidence to support selection (F_ST_, Tajima's D, and Fay and Wu's H) are all better placed to detect evidence of selection in the more distant past, well before the time at which the LCT gene was subjected to selective pressure. It is perhaps not surprising, therefore, that only the linkage disequilibrium-based methods (iHS and Rsb) provide any evidence of selection in this region.

As part of providing access to this computational workflow, it is important to mention the caveats associated with performing selection analyses. Reviews by Nielsen (2005) and Vitti et al. (2013) provide an excellent overview of these and other issues associated with the detection of evidence of selection using genetic data. All of the tools implemented in this pipeline are designed to elucidate patterns of genotypic variation that are consistent with the presence of selective pressure at some time in the past. However, even when such patterns are identified, there is no guarantee that they are the result of selection, rather than other unrelated ancestral events. For example, Tajima's D is known to be sensitive to population growth (Simonsen et al., 1995), whereas methods that assess changes in linkage disequilibrium and/or haplotype frequencies can be influenced by differences in recombination rates across the genome (Nielsen, 2005). Even in the case where selective pressure has led to changes in haplotype frequencies, it may not be possible to identify the type of selection involved. For example, positive selection (e.g., via hard or soft sweeps) may leave a genomic footprint that is indistinguishable from that created by background selection against deleterious mutations (Vitti et al., 2013).

There are a number of improvements that could be made to this workflow. In terms of the various measures of selection that we have employed, we note that calculation of the F_ST_ statistic is dependent on window size and step size, while calculation of Tajima's D statistic is dependent on window size. Ongoing work will examine how best to implement these methods on multiple scales, and allow the results to be combined. Incorporating a measure of the statistical significance of the F_ST_ statistic would also be an improvement, along with appropriate adjustment for multiple hypothesis testing. Similarly, our pipeline could be extended to incorporate probabilistic measures of genotype, particularly relevant for modern genotyping-by-sequencing (GBS) technologies (e.g., Elshire et al., 2011; Majewski et al., 2011) where there is uncertainty in genotype calls (Li, 2011; Li et al., 2011), and for situations where SNP selection methods result in ascertainment bias (Ramírez-Soriano and Nielsen, 2009).

In addition to improving the algorithmic aspects of the pipeline, additional benefit could be gained through the inclusion of support for indel variants. Currently calculation of Fay and Wu's H, iHS and Tajima's D are not carried out for indels. Adding support for this feature would be difficult for human analyses involving iHS and Fay and Wu's H, as the available ancestral FASTA files do not contain any indel information. The inclusion of indels in the Tajima's D calculations is possible, however, and would require a reorganization of the pipeline to ensure indels are preserved until the point at which the D statistic is generated.

The pipeline is also flexible regarding input data types and biological contexts. The entrée into this pipeline is via VCF formatted files, and it can therefore be used to analyse whole genome (re)sequence, transcriptome-derived data, exomes or specific gene candidates of interest on very large samples. More recently, several more computationally-intensive analytical methods have been developed (e.g., Grossman et al., 2010; Ronen et al., 2013) which could potentially be integrated into workflows such as those presented here.

Despite the potential for ongoing enhancement, we believe that in its current state this workflow provides researchers with a valuable tool for investigating selection within a collection of individuals for which high-density genotype data are available, and we hope that the research community is able to make good use of these tools. To that end we have made the pipeline software publicly available as a GitHub repository at: https://github.com/smilefreak/selectionTools

The repository includes an automated installation script, and a detailed manual containing an example analysis that can be followed by new users. The pipeline version corresponding to this publication is 1.0. As additions and refinements are made, these changes will be versioned and commented. However, using the functionality of GitHub, researchers will always be able to access the original published versions of the scripts that are referred to here.

### Conflict of interest statement

The authors declare that the research was conducted in the absence of any commercial or financial relationships that could be construed as a potential conflict of interest.

## Supplementary material

Figure S1 **Plot of F_ST_ values across chromosome 2 (whole chromosome, and the region around the LCT gene) based on 1000 Genomes Project data for the CEU and YRI populations**. A 3 Kbp window was used for calculations. Blue vertical lines/boxes on the plots indicate the location of the LCT gene, and the red horizontal lines denote the mean plus three standard deviations for all the F_ST_ window calculations. Although there is an indication from the whole chromosome plot that regions of this chromosome may have been under selection, there is little support for this in the region of the LCT gene.

Figure S2 **Plot of Tajima's D values across chromosome 2 (whole chromosome, and the region around the LCT gene) based on 1000 Genomes Project data for the CEU and YRI populations**. A 3 Kbp window was used for calculations. Blue vertical lines/boxes on the plots indicate the location of the LCT gene. Based on the values of the statistic around the LCT gene, there is no evidence of this region having been under selection in either the CEU or YRI populations.

Figure S3 **Plot of Fay and Wu's H values across chromosome 2 (whole chromosome, and region around LCT gene) based on 1000 Genomes Project data for the CEU and YRI populations**. While there appears to be some evidence for regions of the chromosome having been subjected to selective pressure, when the area around the LCT gene is examined, for both populations the values of H in this region are unremarkable relative to those in the remainder of the chromosome.
