# Supplementary figures and images for: A bioinformatics workflow for detecting signatures of selection in genomic data
Source: Front Genet. 2014 Aug 26;5:293. doi: 10.3389/fgene.2014.00293 (PMC4144660; doi:10.3389/fgene.2014.00293)

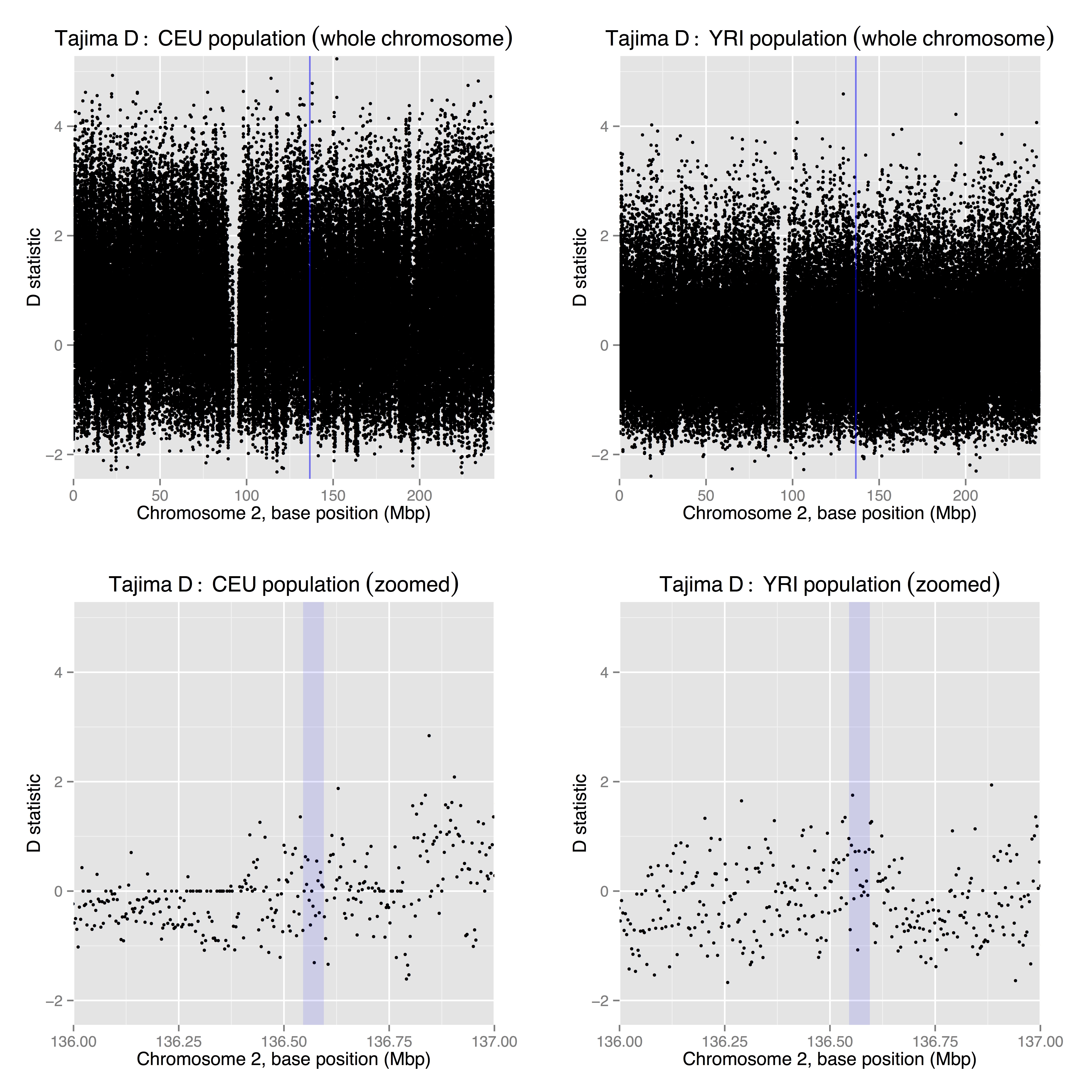

Supplement: Figure S1 — Plot of FST values across chromosome 2 (whole chromosome, and the region around the LCT gene) based on 1000 Genomes Project data for the CEU and YRI populations. A 3 Kbp window was used for calculations. Blue vertical lines/boxes on the plots indicate the location of the LCT gene, and the red horizontal lines denote the mean plus three standard deviations for all the FST window calculations. Although there is an indication from the whole chromosome plot that regions of this chromosome may have been under selection, there is little support for this in the region of the LCT gene. [file DataSheet1.ZIP › Figure S2.JPEG]

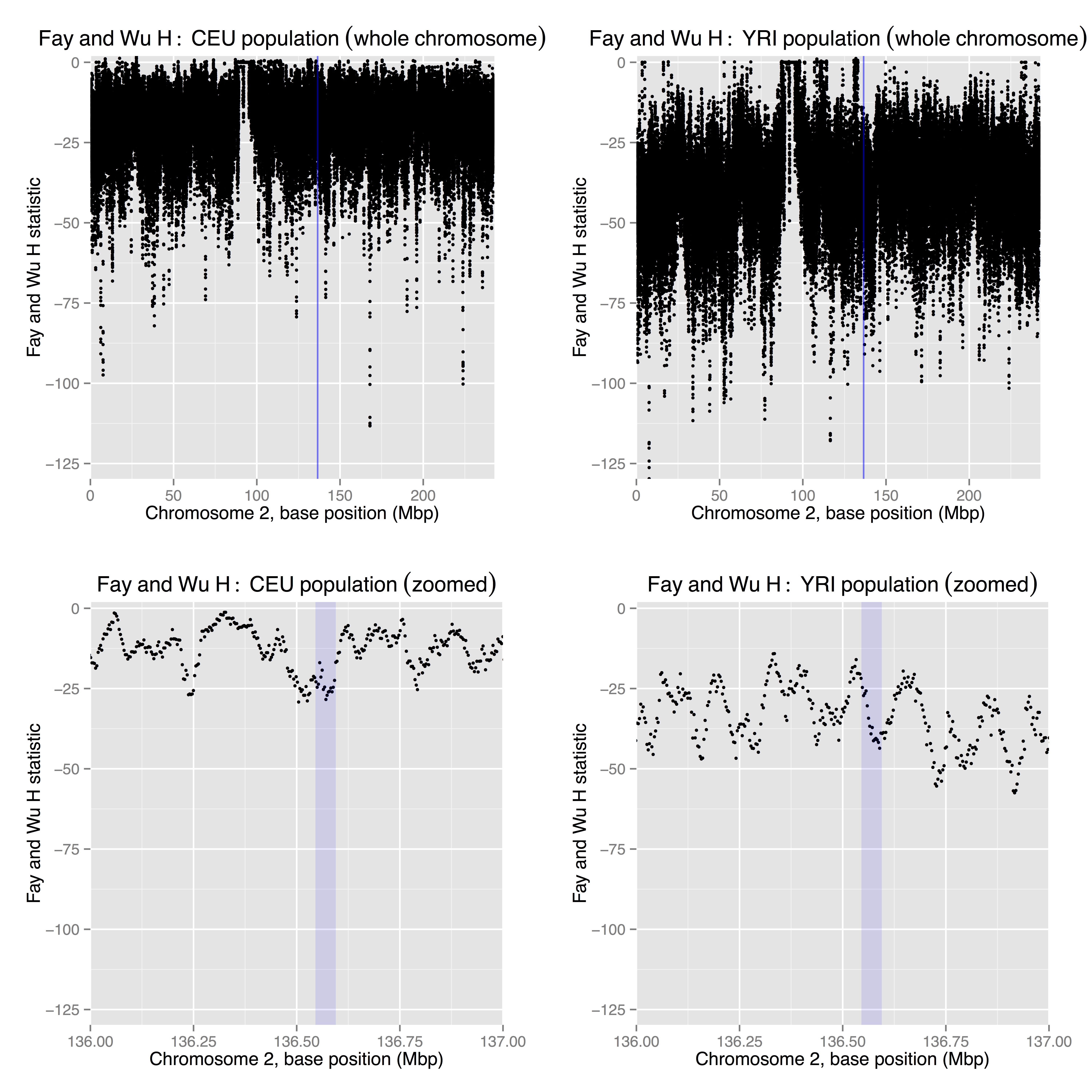

Supplement: Figure S1 — Plot of FST values across chromosome 2 (whole chromosome, and the region around the LCT gene) based on 1000 Genomes Project data for the CEU and YRI populations. A 3 Kbp window was used for calculations. Blue vertical lines/boxes on the plots indicate the location of the LCT gene, and the red horizontal lines denote the mean plus three standard deviations for all the FST window calculations. Although there is an indication from the whole chromosome plot that regions of this chromosome may have been under selection, there is little support for this in the region of the LCT gene. [file DataSheet1.ZIP › Figure S3.JPEG]

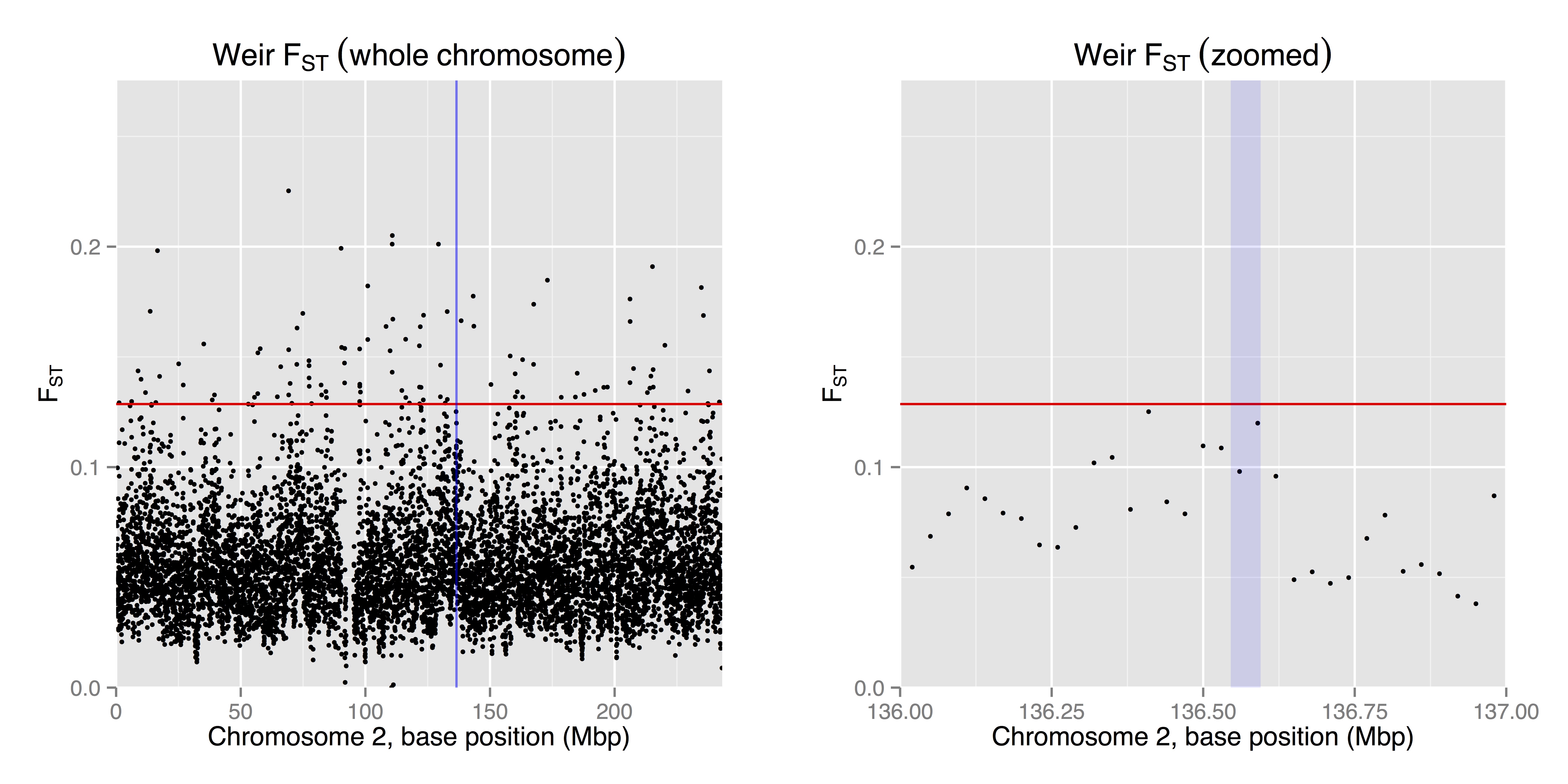

Supplement: Figure S1 — Plot of FST values across chromosome 2 (whole chromosome, and the region around the LCT gene) based on 1000 Genomes Project data for the CEU and YRI populations. A 3 Kbp window was used for calculations. Blue vertical lines/boxes on the plots indicate the location of the LCT gene, and the red horizontal lines denote the mean plus three standard deviations for all the FST window calculations. Although there is an indication from the whole chromosome plot that regions of this chromosome may have been under selection, there is little support for this in the region of the LCT gene. [file DataSheet1.ZIP › Figure S1.JPEG]
